# *Streptococcus gallolyticus* Increases Expression and Activity of Aryl Hydrocarbon Receptor-Dependent CYP1 Biotransformation Capacity in Colorectal Epithelial Cells

**DOI:** 10.3389/fcimb.2021.740704

**Published:** 2021-10-27

**Authors:** Rahwa Taddese, Rian Roelofs, Derk Draper, Xinqun Wu, Shaoguang Wu, Dorine W. Swinkels, Harold Tjalsma, Annemarie Boleij

**Affiliations:** ^1^Department of Pathology, Nijmegen Institute for Molecular Life Sciences (RIMLS), Radboud University Medical Centre (Radboudumc), Nijmegen, Netherlands; ^2^Laboratory Medicine, Nijmegen Institute for Molecular Life Sciences (RIMLS), Radboud University Medical Centre (Radboudumc), Nijmegen, Netherlands; ^3^Department of Medicine, Division of Infectious Diseases, Johns Hopkins University, Baltimore, MD, United States

**Keywords:** *Streptococcus gallolyticus*, colorectal cancer, gut microbiota, biotransformation, Aryl hydrocarbon (Ah) receptor

## Abstract

**Objective:**

The opportunistic pathogen *Streptococcus gallolyticus* is one of the few intestinal bacteria that has been consistently linked to colorectal cancer (CRC). This study aimed to identify novel *S. gallolyticus*-induced pathways in colon epithelial cells that could further explain how *S. gallolyticus* contributes to CRC development.

**Design and Results:**

Transcription profiling of *in vitro* cultured CRC cells that were exposed to *S. gallolyticus* revealed the specific induction of oxidoreductase pathways. Most prominently, *CYP1A* and *ALDH1* genes that encode phase I biotransformation enzymes were responsible for the detoxification or bio-activation of toxic compounds. A common feature is that these enzymes are induced through the Aryl hydrocarbon receptor (AhR). Using the specific inhibitor CH223191, we showed that the induction of *CYP1A* was dependent on the AhR both *in vitro* using multiple CRC cell lines as *in vivo* using wild-type C57bl6 mice colonized with *S. gallolyticus*. Furthermore, we showed that CYP1 could also be induced by other intestinal bacteria and that a yet unidentified diffusible factor from the *S. galloltyicus* secretome (SGS) induces CYP1A enzyme activity in an AhR-dependent manner. Importantly, priming CRC cells with SGS increased the DNA damaging effect of the polycyclic aromatic hydrocarbon 3-methylcholanthrene.

**Conclusion:**

This study shows that gut bacteria have the potential to modulate the expression of biotransformation pathways in colonic epithelial cells in an AhR-dependent manner. This offers a novel theory on the contribution of intestinal bacteria to the etiology of CRC by modifying the capacity of intestinal epithelial or (pre-)cancerous cells to (de)toxify dietary components, which could alter intestinal susceptibility to DNA damaging events.

## Introduction

The resident gut microbiota is essential for human intestinal health and prevents the invasion of pathogens by providing colonization resistance and nutrient competition (Vollaard and Clasener, 1994; Hooper et al., 2002). The human epithelium itself wards of infections by the excretion of a continuous protective mucus layer and antimicrobials and by tightly sealing the paracellular space between adjacent cells (Matsuo et al., 1997; Chichlowski and Hale, 2008). However, in case of intestinal diseases such as inflammatory bowel disease (IBD) and colorectal cancer (CRC), these protective mechanisms are impaired, and the bowel wall becomes prone to bacterial infiltration, rendering the host more susceptible to opportunistic bacterial infections (Aksoy and Akinci, 2004; Stecher and Hardt, 2008).

One of the intestinal bacteria that has been consistently linked to human CRC is the opportunistic pathogen *Streptococcus gallolyticus* (previously known as *Streptococcus bovis* biotype I). Multiple studies have documented that 33%–100% of *S. gallolyticus-*infected patients have concomitant adenomas and carcinomas, which largely exceed the CRC rates reported in the general population (Lieberman et al., 2000; Corredoira et al., 2005; Boleij et al., 2011). Notably, nearly all of these patients did not present with gastrointestinal signs or symptoms, and the (pre-)cancerous lesions were thus solely detected based on the clinical infection with *S. gallolyticus*.

It is suggested that CRC or advanced adenomas provide a specific niche for *S. gallolyticus*. One of the described mechanisms is the collagen-binding ability of *S. gallolyticus*, which potentially contributes to the specific colonization of malignant colonic sites (Boleij et al., 2011). Furthermore, colonic tumor cell metabolites could facilitate the survival of *S. gallolyticus*, favoring its local outgrowth (Boleij et al., 2012). More recent insight shows specific increase in colonization in tumor-bearing mice, which is most likely due to an increase in the presence of secondary bile acids, resulting in the production of gallocin by *S. gallolyticus* reducing the presence of other enterococci, e.g., *Enterococcus faecalis*, and favoring its colonization (Aymeric et al., 2018; Pasquereau-Kotula et al., 2018). However, the specific binding to CRC tissue is controversial and debated. Although DNA-based approaches in three independent studies show *S. gallolyticus* colonization ranging from 0% to 2% in controls, 47% in normal tissue of cancer patients, and 3%–74% in tumor tissues (Abdulamir et al., 2010; Andres-Franch et al., 2017; Kumar et al., 2018), two other studies reported no significant difference between healthy and CRC patients (Boltin et al., 2015; Viljoen et al., 2015). In addition, no specific tumor cell enrichment was observed in tumor-bearing mice (Aymeric et al., 2018).

Although an incidental relationship between *S. gallolyticus* and CRC seems plausible, it still remains to be determined to which extent *S. gallolyticus* may also play a contributory role in the carcinogenesis process itself. It was already shown that the related bacterium *Streptococcus infantarius* strain NCTC8133 (now designated as *Streptococcus equinus*) increased the amount of aberrant crypt foci and adenomas in an azoxymethane (AOM)-induced rat model (Ellmerich et al., 2000) and increased COX-2 expression (Biarc et al., 2004). Similar results have been obtained for *S. gallolyticus* using an AOM-induced mouse model for CRC. This was corroborated by *in vitro* data that showed *S. gallolyticus* subsp. *gallolyticus* increases cell proliferation in HT29, HCT116, and LoVo cells, by increasing *beta*-catenin translocation to the nucleus and c-myc expression. Importantly, the latter was not observed with *S. gallolyticus* subsp. *macedonicus* and *S. gallolyticus* subsp. *pasteurianus* (Kumar et al., 2017). Our recent report shows that cell proliferation increase or decrease depends on the *S. bovis* substrain and the CRC cell lines employed (Taddese et al., 2020). This shows a complex relationship not only at strain level but also between CRC cells from different origin.

The aim of the current research was focused on the identification of *S. gallolyticus* subsp. *gallolyticus*-induced epithelial pathways that can, on the long-term, contribute to carcinogenesis. Our results show that a factor from *S. galloltyicus* that is also present in the *S. gallolyticus* secretome (SGS), induced cytochrome P450 (CYP)1 persistently through the Aryl hydrocarbon receptor (AhR) in four different colon adenocarcinoma cells. These expression data were confirmed in a mouse model, and subsequently, we show that SGS could increase the DNA damaging effect of the polycyclic aromatic hydrocarbon 3-methylcholanthrene *in vitro*. The AhR activation and CYP1 induction appeared to be *S. bovis* strain dependent and may potentially also be induced by other intestinal bacteria.

## Materials and Methods

### Cell Culture

The colorectal adenocarcinoma cell lines (CRC cells) HT-29, SW480, HCT116, and Caco-2 (www.atcc.org) were cultured in Dulbecco’s modified Eagle’s medium (DMEM, Lonza) supplemented with 10% fetal calf serum (FCS), 20 mM HEPES, 2 mM L-glutamine, and 1× non-essential amino acids (Gibco) at 37°C/5% CO_2_. For gene-expression analysis, cells were serum starved to 1% FCS for 24 h before co-culturing with bacterial cells. These culturing conditions were used unless stated otherwise.

### Bacterial Strains

The following *Streptococcus bovis* strains were used: *S. gallolyticus* subsp. *gallolyticus* strains UCN34 (Rusniok et al., 2010), NTB1 (Boleij et al., 2011), 1293, 1294 (Tripodi et al., 2005), and NTB12; *Streptococcus infantarius* NCTC8133 (Biarc et al., 2004) (NZ_LR594042.1); *Streptococcus lutetiensis* NTB2; *S. gallolyticus* subsp. *macedonicus* strains CIP105685T, 19AS, and ACA-DC205; and *S. gallolyticus* subsp. *pasteurianus* strains 992 and NTB7. A phylogenetic tree of these *S. bovis* strains was published in Taddese et al., 2020 (Taddese et al., 2020). Other bacterial strains were *E. faecalis* 19433 (www.atcc.org), *Enterobacter cloacae* NTB9, *Staphylococcus lugdunensis* NTB8, *Salmonella typhimurium* NTB6, *Escherichia coli* Nissle 1917, and *E. coli* NTB5 (Boleij et al., 2011). All strains were grown on Columbia blood agar or in brain heart infusion (BHI) broth (Difco) supplemented with 1% glucose at 37°C/5% CO_2_. *E. coli* was grown at 200 rounds per minute (rpm).

### Cell Proliferation Using MTT Assay

For MTT assays, optimal cell seeding in 96-well plates (Greiner Bio-One, Austria) was defined at 5,000 cells/well for Caco-2, 1,500 cells/well for HCT116, 10,000 cells/well for HT29, and 6,000 cells/well for SW480 to allow for 72 h growth observation. Cells were incubated overnight to attach before addition of bacterial secretomes in a fourfold dilution in DMEM supplemented with 10% FCS. Secretomes were collected as follows. After culturing in BHI broth, bacteria were centrifuged at 4,700 rpm for 20 min and filter sterilized using 0.2-µm filters (Sigma-Aldrich, USA). Molecules larger than 10 kDa were concentrated using Amicon ultra-15 centrifugal filters (Merck Millipore, Merck, USA). Concentrated secretomes were frozen at −80°C until further use. MTT-assays were performed as described previously, and MTT assay was performed after 24, 48, and 72 h. Metabolic activity of CRC cells measured by MTT assay was used as a measure for cell growth. All experiments were performed in quadruplicate. Area under the curve was calculated for each condition (strain) and compared to cells incubated with cell culture media containing BHI broth as control using independent sample t-test in GraphPad Prism 9.

### Microarray Analysis

HT-29 cells were grown to confluence in T75 flasks and subsequently incubated with or without *S. gallolyticus* [multiplicity of infection (MOI) of 20] in two duplicate experiments. After 2 h, the culture medium was refreshed, and incubation was prolonged for another 2 h. This 4-h incubation period was chosen for microarray analysis because induction of the COX-2 marker for chronic inflammation and CRC progression (Ellmerich et al., 2000) could be determined under these conditions. Next, HT-29 monolayers were washed three times with prewarmed PBS after which RNA was isolated according to the Qiagen protocol (Qiagen RNeasy kit) with on-column DNA digestion. RNA quantity was measured with Nano-drop (Thermo Scientific, USA), and RNA quality was checked with the Bio-analyzer 2000 (Agilent Technologies, USA). Samples with RNA integrity scores ≥9.0 were approved for microarray analysis.

Gene expression profiling was performed using Affymetrix GeneChip Human Gene 1.0 ST arrays, representing all known human genes (Affymetrix Inc., Santa Clara, CA, USA). The Affymetrix GeneChip Whole Transcript Sense Target Labeling Assay was used to generate amplified and biotinylated sense-strands DNA targets from the entire expressed genome (1.0 µg of total RNA). The manufacturer’s manual was followed for the hybridization, washing, and scanning steps (version 4, P/N 701880 Rev. 4). Arrays were hybridized by rotating them at 60 rpm in the Affymetrix GeneChip hybridization oven at 45°C for 17 h. After hybridization, the arrays were washed in the Affymetrix GeneChip Fluidics station FS 450. Arrays were scanned using the Affymetrix GeneChip scanner 3000 7G system.

The Affymetrix CEL files were first imported into Affymetrix Expression Console version 1.1 where control probes were extracted using the default robust multichip averaging (RMA) algorithm in order to perform quality analysis checks. The Area Under the Curve (AUC) of the Receiver Operator Characteristic was calculated using the positive and negative control probes. All arrays had an AUC score above the empirically defined threshold of 0.85 indicating a good separation of the positive controls from the negative controls.

Subsequently the CEL files were imported into Partek^®^ (Partek^®^ Genomic Suite software, version 6.4 Copyright^©^ 2008 Partek Inc., St. Louis, MO, USA) where only core exons were extracted and normalized using the RMA algorithm with GC background correction. Core transcript summaries were calculated using the mean intensities of the corresponding probesets. The correspondence of the replicate samples was confirmed using principle component analysis (PCA) and Pearson correlation analysis. After grouping the samples by cell type, an ANOVA was performed on the log2 intensities, and contrast p-values were calculated for all pair-wise comparisons between the sample groups. Genes with a fold change of at least 1.5 and a p-value below 0.05 were selected for further evaluation. Raw data files are deposited in the Gene Expression Omnibus (GEO) database, available through: http://www.ncbi.nlm.nih.gov/geo/query/acc.cgi?token=jtcvzsakgwaiibo&acc=GSE29295.

### Quantitative Real-Time PCR

For determination of *CYP1A1* and *ALDH1A3* gene expression in time-series experiments, CRC cell lines were incubated with *S. gallolyticus* (MOI, 20) for the indicated time periods. To determine concentration dependence and additive effects of 1 and 10 nM 3-methylcholanthrene (3MC; Sigma) on *CYP* gene expression, cells were incubated for 4 h with the indicated bacterial MOIs. To determine AhR-dependent *CYP* gene expression, CRC cells were incubated in the presence of the AhR-antagonist CH-223191 (3 µM; Calbiochem) for 1 h and subsequently incubated with *S. gallolyticus* (MOI, 20) and/or 1 nM 3MC. These relatively low concentrations of 3MC (1 and 10 nM) were used to avoid saturation of CYP1A1 induction by 3MC, which allowed to determine the additive effect of *S. gallolyticus* on CYP1A1 expression under these conditions. To determine the effect of bacteria-associated factors on *CYP* gene expression, *S. gallolyticus*, *E. faecalis*, or *E. coli* were cocultured with Caco-2 cells for 6 h. Then, the supernatants were spun for 10 min at 4,000 *g* and subsequently passed through a 0.2-µm filter to remove bacteria. Next, fresh serum-starved Caco-2 cells (24 h) were incubated with the secretomes for the indicated time periods. Caco-2 and HT-29 cells were incubated with the bacterial strains at a MOI of 20 for 2 and 4 h to determine the differences between CYP1A1 induction for the different *S. bovis* subspecies in comparison with the Gram-positive bacterium *E. faecalis* and the Gram-negative bacterium *E. coli*. The above-described interventions were all performed in at least two replicate experiments. CRC cells were disrupted in RLT-lysis buffer, and RNA was extracted according to the Qiagen protocol (RNeasy Mini-Kit). Next, Iscript Reverse Transcriptase PCR (Bio-Rad) was performed to synthesize 1 µg of cDNA under the following conditions: 5 min at 25°C, 30 min at 42°C, and 5 min at 85°C. Expression of the genes listed in **Supplementary Table S1** was compared to the expression of the household gene glyceraldehyde-3-phosphate dehydrogenase (GAPDH) (4310884E gene expression assay; Applied Biosystems) using the following quantitative real-time PCR (qPCR) protocol: 2 min at 50°C, 10 min at 95°C, and 40 cycles of 15 s at 95°C and 60 s at 60°C (7900 HT, Applied Biosystems). All genes measured with custom primers (Biolegio) were analyzed with SYBR green Mastermix (Applied Biosystems), and all gene-expression assays were analyzed with Universal Mastermix (Applied Biosystems). The relative quantity (RQ) values were calculated *via* the ΔΔCt method (Pfaffl, 2001) using SDS 2.2.1 software. The log2 values of the ΔΔCt values were plotted on a linear scale, whereby the 0 level represents no expression change. For all data with two grouping variables, two-way ANOVAs were performed, and for data with one grouping variable, one-way ANOVA or Student’s t-test were performed in GraphPad Prim 5. Differences were considered significant below p-value of 0.05.

### Western Blotting

Expression of *CYP1A1* was evaluated at the protein level in Caco-2 cells, as these cells are best suited to measure CYP1A1 at the protein level (Meunier et al., 1995). However, it should be noted that also in this case, CYP1A1 levels are below the level of detection without addition of specific stimulating agents (Rosenberg and Leff, 1993). The experiments of this study were further challenged by the fact that only viable bacterium strongly stimulates CYP1A1, whereas prolonged coincubations with viable bacteria resulted in the death of Caco-2 cells. To overcome these challenges, confluent Caco-2 monolayers were incubated with 1 µM 3MC or cocultured with *S. gallolyticus* at a MOI of 50. The use of this high 3MC concentration and high MOI allowed a rapid and relatively strong induction of CYP1A1, while cellular damage by bacterial proliferation at ≤6 h time points was minimized. After 6 h of incubation, Caco-2 cells were refreshed with medium containing 10 µg/ml chloramphenicol to block further bacterial growth for prolonged incubations. After 6, 9, and 12 h of incubation, cells were dislocated in PBS-EDTA and disrupted in lysis buffer (50 mM HEPES, 0.5 M NaCl, 1.5 mM MgCl_2_, 1 mM EDTA, 10% glycerol, and 1% Trition-X100) (Anwar-Mohamed and El-Kadi, 2009). As control of induction and to test several different CYP1A antibodies (**Supplementary Information**) Caco-2 cells were incubated with 1 μM 3MC for 20 h or non-treated and subsequently lysed. Lysates were vortexed every 10 min during incubation on ice for 1 h. Next, samples were spun at 12,000 *g* for 10 min and stored at −20°C until further analysis. Fifty micrograms of protein in sodium dodecyl sulfate (SDS) sample buffer (50 mM Tris–HCl, pH 6.0, 2% SDS, 5% β-mercaptoethanol, and 10% glycerol) was incubated for 5 min at 95°C prior to 12.5% glycine SDS-polyacrylamide gel electrophoresis (SDS-PAGE) (Towbin et al., 1979). Proteins were transferred to polyvinylidene fluoride (PVDF) membranes (Amersham) by Western blotting. Next, membranes were blocked for 1 h in 5% bovine serum albumin (BSA) in Tris-buffered saline supplemented with Tween 20 (0.1%) (TBS-T) and incubated with monoclonal mouse anti-CYP1A1 antibodies (Santa Cruz) diluted 1:500 in TBS-T, monoclonal mouse anti-β-actin antibody (Sigma A5441) diluted 1:25,000 in TBS-T, monoclonal mouse anti-CYP1A1/1A2 antibodies (K06; collection of Dr. W. Peters) diluted 1:800 in TBS-T, or polyclonal rabbit anti-CYP1A1 antibody (Human Biologics Inc.) diluted 1:1,000 in 5% BSA, or polyclonal rabbit anti CYP1A1/1A2 antibody (Human Biologics Inc.) diluted 1:800 in 5% BSA. Bound antibodies were visualized with the ECL detection system (Amersham) using antimouse immunoglobulin G (IgG) horseradish peroxidase (HRP) conjugates diluted 1:50,000 or antirabbit IgG HRP conjugates diluted 1:50,000 in 5% milk in TBS-T (Jackson ImmunoResearch).

### 7-Ethoxyresorufin-O-Deethylase Enzyme Activity Assay

After incubation for 6, 9, and 12 h, Caco-2 cells were washed with ice-cold PBS and lysed in 0.1M potassium phosphate buffer (0.1M dipotassium phosphate, 0.1M EDTA, and 20% glycerol, pH 7.4). Note that viable bacteria were removed after the 6-h time point to avoid premature cell death by excessive bacterial proliferation as described in the previous section. Cells were disrupted by mechanical shearing on ice (crude cell lysates) and stored at −80°C until use. Protein concentrations were determined with the Bradford protein assay following the manufacturers’ description (BioRad). Crude cell lysates were diluted to 100 µg/ml protein in assay buffer (0.1M dipotassium phosphate and 0.1M EDTA, pH 7.4), and 4 µM ethoxyresorufin (Molecular Probes) was added. Samples were prewarmed at 37°C before addition of 10 µM reduced nicotinamide adenine dinucleotide phosphate (NADPH) (Sigma). The conversion of ethoxyresorufin (non-fluorescent) into resorufin (fluorescent) is catalyzed by CYP1A1 and requires the presence of NADPH. The reaction was started by the addition of NADPH and resorufin production in the samples was measured during a 10-min time period on a Shimadzu RF-5000 spectrofluorometer at an excitation of 550 nm and emission of 580 nm. Known concentrations of resorufin (Molecular Probes) were used to create a calibration curve to calculate the amount of resorufin formed from ethoxyresorufin, catalyzed by the CYP1A1 enzyme (Yu et al., 2001). To compare individual samples, the amount of resorufin produced per minute was related to the protein concentration in the sample (resorufin/min·mg). The amount of resorufin produced per minute per milligram is a measure of the CYP1A1 activity in the sample. Two-way ANOVA statistics was used to determine significant changes.

### COMET Assay

The additional effects of bacterial products on DNA damage by 0.1 µM 3MC were measured using the COMET assay (Trevigen). Secretomes of *S. gallolyticus*, *E. faecalis*, and *E. coli* were incubated with Caco-2 cells in quadruplicate. After priming Caco-2 cells for 6 h with these secretomes, 0.1 µM 3MC was added, and incubation was prolonged for 18 h. This relatively low concentration of 3MC was used to allow assessment of a possible additive effect of bacterial stimulation on 3MC toxicity. Higher concentrations could mask such an additive effect due to saturation of CYP1A1 induction by 3MC itself. H_2_O_2_ (100 µM) was used as positive control for DNA damage. After incubation of Caco-2 cells with the supernatants, 3MC or H_2_O_2_, cells were washed with warm PBS, harvested by trypsin treatment and spun at 200 g for 5 min. Cells were counted, and 8,000 cells per condition were washed with Mg- and Ca-free ice-cold PBS. Next, cells were dissolved in low-melting agarose and mounted on COMET slides. After 30 min at 4°C in the dark to solidify the agarose, the immobilized cells were disrupted in Trevigen lysis solution for 60 min at 4°C. After lyses of the cells in the solidified agarose, DNA was unwound by alkaline treatment (pH >13) for 30 min. Subsequent electrophoresis for 30 min at 25 V was employed to yield migration of DNA in the agarose. In this assay, the migration distance of DNA is a measure of the amount of DNA damage under a certain condition. After electrophoresis, COMET slides were washed in 0.4M Tris–HCl (pH 7.5) and fixed for 10 min in absolute alcohol. DNA damage (observed as COMET-like structures) was visualized by SYBRgreen staining and fluorescence microscopy (Leica). A total of 100 COMETs per condition were independently scored by two researchers that were uninformed about the experimental conditions, following the methodology of Collins et al. (Collins, 2004). DNA damage was calculated as reference to the positive H_2_O_2_ control. One-way ANOVA in GraphPad Prism 4.00 was performed to determine significance of the results (p < 0.05).

### Mice Colonization With *S. gallolyticus*

The mouse strains used in this study were C57bl6 purchased from Jackson Laboratory (Bar Harbor, ME, USA) or obtained as littermates of in-house breeding. We administered kanamycin (1 g/L) and doxycyclin (100 mg/L) for 5 days in drinking water, followed by oral inoculation with the *S. gallolyticus* strain UCN34 (~5 × 10^7^ and 5 × 10^8^ bacteria in PBS) or PBS alone (sham control) in mice at 4 weeks of age. Simultaneously with antibiotics pretreatment, mice were orally gavaged with the AhR-inhibitor CH223191 (10 µg/g/day) dissolved in corn oil (vehicle) or with vehicle alone, continued until sacrifice at day 7 postinoculation of *S. gallolyticus*. Mice were randomly placed in groups, making sure that there was no cage effects for mice experiments. Sham mice were housed separately from *S. gallolyticus* colonized mice. We quantified fecal bacterial colonization as colony-forming units (CFUs) per gram stool on BHI agar supplemented with 5 µg/ml doxycyclin. Single colonies were boiled in 50 μl dH2O, and *S. gallolyticus* colonization was confirmed with SodA PCR (SodA d1- CCTTATGCATATGATGCTCTTGAGCC, SodA d2-AGATAGTAAGCGTGTTCCCAAACGTC, 488 bp product; DNA was denatured at 95°C for 10 min followed by 40 cycles at 94°C for 30 s, 56°C for 35 s, and 72°C for 72 s, and subsequent elongation for 7 min at 72°C) (Poyart et al., 2002). At experimental time points, we harvested one piece each of the cecum in Trizol reagent for RNA analysis. qPCR was performed on RNA extracted from Trizol using TaqMan gene expression assays for CYP1A1 (Mm00487218_m1), IL4 (Mm00445259_m1), AhR (Mm00478932_m1), and PTGS2 (Mm00478374_m1) relative to 18s (4318839) and standard operation conditions on 7500 fast system (Applied Biosystems). The colons were Swiss rolled, paraffin embedded, and subsequently sectioned at 4 μm for H&E/periodic acid–Schiff (PAS) staining or IHC ki67 analysis. Histology was reviewed by an expert GI pathologist. All mice were kept in specific pathogen-free (SPF) conditions prior to *S. gallolyticus* colonization in the Johns Hopkins University (JHU) animal facility. The mouse protocols were approved by the Johns Hopkins University Animal Care and Use Committee in accordance with the Association for Assessment and Accreditation of Laboratory Animal Care International.

### *S. gallolyticus* ELISA

To measure serum response to *S. gallolyticus*, 96-well plates were coated with 50 µg/ml collagen IV from rat tail in 0.02M acetic acid for 1 h. Plates were washed twice in PBS. Next, plates were coated with 1 × 10^9^ *S. gallolyticus* UCN34 per well o/n in PBS at 37°C. Plates were fixed in 4% paraformaldehyde for 1 h at room temperature and stored at 4°C until further use. Wells were blocked with 3% skim milk in PBS–0.5% Tween-20 (PBS-T). Serum was diluted in 3% skim milk in PBS-T 1:50 and incubated for 1.5 h at room temperature with gentle shaking. A serum-free control was used as reference together with non-infected mouse serum. Plates were washed four times in PBS-T for 5 min and subsequently incubated with antimouse-HRP in 3% skim milk in PBS-T 1:5,000 for 1 h at room temperature. Plates were washed four times for 5 min in PBS-T and developed with 100 µl TMB substrate in 5–10 min. The reaction was stop with 50 µl 2N H_2_SO_4_. The OD was measured at 450 nm in a BioRad plate reader with reference filter at 655 nm within 30 min after stopping the reaction.

## Results

### Microarray Analysis Uncovers Induction of Host Oxidoreductase Gene Expression by *S. gallolyticus*

**Figure 1:**
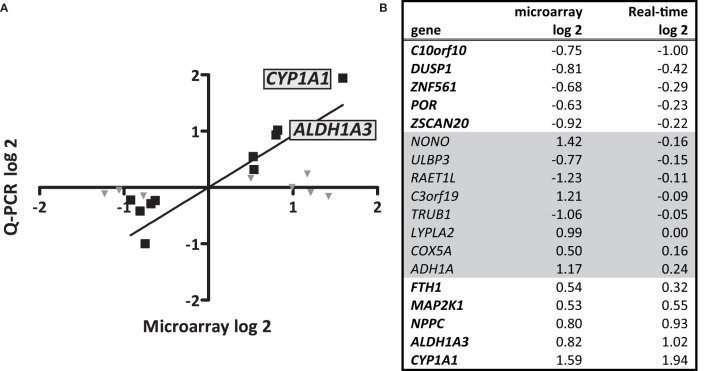
Microarray data and validation. Microarray analysis was performed to profile *S. gallolyticus* UCN34-induced pathways that could be involved in CRC. **(A)** The upregulation of 18 selected genes was validated by quantitative real-time PCR (qPCR) analysis. The positions of the most significantly upregulated genes CYP1A1 and ALDH1A3 are indicated. **(B)** The corresponding log 2 values of microarray and qPCR are listed for each gene. The 10 genes that showed similar effects in microarray and qPCR [Pearson correlation (r = 0.94; p < 0.001)] are **(B)** printed in bold and **(A)** marked by black squares. Gene expression changes that could not be validated by qPCR are **(B)** printed in gray and **(A)** marked by gray triangles.

To unravel the effects of *S. gallolyticus* on gene expression of CRC cells, a transcriptome analysis was performed 4 h after coculturing of *S. gallolyticus* UCN34 with HT29 CRC cells. Microarray analysis showed a total of 44 significantly differentially expressed genes upon *S. gallolyticus* exposure (**Table 1**), which was a surprisingly low number in comparison to about 150 differentially expressed genes upon coincubation with other non-pathogenic bacteria under the same conditions (GEO-GSE29295). Strikingly, as much as nine of these *S. gallolyticus*-regulated genes appeared to belong to oxidoreductase pathways (**Table 1** in bold), including *CYP1A1, ADH1A*, and *ALDH1A3* (**Supplementary Figure S1**). For validation, 18 (out of 44) genes with normalized expression levels >6 on the used Affymetrix GeneChip were selected. The corresponding log-2 values for microarray and qPCR of 10 of these genes showed similar levels by both methods as predicted by Pearson correlation (r = 0.94; p < 0.001) (**Figures 1A, B**). In line with microarray results, *CYP1A1* (log 2 RQ, 1.94) and *ALDH1A3* (log 2 RQ, 1.02) were also found to be the most significant differentially expressed genes by qPCR (**Figure 1** and **Supplementary Figure S2**). Taken together, these data imply that *S. gallolyticus* induces the expression of *CYP1A1* and *ALDH1A3*. Interestingly, the corresponding enzyme activities of these genes are involved in biotransformation of drugs and food components and are best known for their clearing function in the liver (Nebert and Dalton, 2006). As the expression of these enzymes is elevated during CRC progression (McKay et al., 1993; Ding and Kaminsky, 2003; Huang et al., 2009), and thereby could be related to colon carcinogenesis, we decided to further focus on these genes.

**Table 1:** Microarray analysis significantly differentially expressed genes in HT29 CRC cells.

Reference	Gene	FC (p < 0.05)
**Upregulated genes**		
NM_000499	**Cytochrome P450, family 1, subfamily A, polypeptide 1 (CYP1A1)**	3.01
NM_007363	Non-POU domain containing, octamer-binding (NONO)	2.68
NM_016474	Chromosome 3 open reading frame 19 (C3orf19)	2.31
NM_000667	**Alcohol dehydrogenase 1A (ADH1A)**	2.25
NM_007260	Lysophospholipase II (LYPLA2)	1.99
NM_012399	Phosphatidylinositol transfer protein, beta (PITPNB)	1.82
NM_000693	**Aldehyde dehydrogenase 1 family, member A3 (ALDH1A3)**	1.77
NM_024409	Natriuretic peptide C (NPPC)	1.75
NM_013252	C-type lectin domain family 5, member A (CLEC5A)	1.54
NM_001083538	POTE ankyrin domain family, member E (POTE2)	1.54
NM_003079	SWI/SNF related regulator of chromatin, member 1 (SMARCE1)	1.5
NM_002308	Lectin, galactoside-binding, soluble, 9 (LGALS9)	1.48
NM_013289	Killer cell immunoglobulin-like receptor (KIR3DL1)	1.46
NM_002032	**Ferritin, heavy polypeptide 1 (FTH1)**	1.45
NM_004891	Mitochondrial ribosomal protein L33 (MRPL33)	1.45
NM_002755	**Mitogen-activated protein kinase kinase 1 (MAP2K1)**	1.44
NM_173359	Eukaryotic translation initiation factor 4E family member 3 (EIF4E3)	1.44
NM_182488	Ubiquitin specific peptidase 12 (USP12)	1.43
NM_005004	**NADH dehydrogenase (ubiquinone) 1 beta subcomplex, 8 (NDUFB8)**	1.42
NM_178433	late cornified envelope 3B (LCE3B)	1.42
NM_004255	**Cytochrome c oxidase subunit Va (COX5A)**	1.42
**Downregulated genes**		
AF280797	Ghrelin opposite strand RNA 2 (non-protein coding) (C3orf42)	0.70
NM_052861	Chromosome 4 open reading frame 42 (C4orf42)	0.70
NM_144712	Solute carrier family 23 (nucleobase transporters) (SLC23A3)	0.69
NM_000437	Platelet-activating factor acetyl hydrolase 2 (PAFAH2)	0.69
BC090889	AHNAK nucleoprotein 2 (AHNAK2)	0.69
NM_001384	DPH2 homolog (DPH2)	0.69
NM_018373	Synaptojanin 2 binding protein (SYNJ2BP)	0.68
NM_002500	Neurogenic differentiation 1 (NEUROD1)	0.66
NM_006147	Interferon regulatory factor 6 (IRF6)	0.65
NM_000941	**P450 (cytochrome) oxidoreductase (POR)**	0.65
NM_001009955	Single-stranded DNA binding protein 3 (SSNP3)	0.64
NM_001930	Deoxyhypusine synthase (DHPS)	0.64
NM_005793	Non-metastatic cells 6, protein (NME6)	0.63
NM_015690	Serine/threonine kinase 36 (STK36)	0.63
NM_152289	Zinc finger protein 561 (ZNF561)	0.62
NM_015911	Zinc finger protein 691 (ZNF691)	0.62
NM_001005749	Glucosidase, beta, acid (GBA)	0.61
NM_007021	Chromosome 10 open reading frame 10 (c10orf10)	0.59
NM_024518	UL16 binding protein 3 (ULBP3)	0.59
NM_004417	**Dual specificity phosphatase 1 (DUSP1)**	0.57
NM_145238	Zinc finger and SCAN domain containing 20 (ZSCAN20)	0.53
NM_139169	TruB pseudouridine (psi) synthase homolog 1 (TRUB1)	0.48
NM_130900	Retinoic acid early transcript 1L (RAET1L)	0.43

A total of 44 genes were significantly up- (21 genes) or downregulated (23 genes) in HT-29 cells after exposure to S. gallolyticus for 4 h; the corresponding fold change (FC) and p-value are listed. Genes printed in bold belong to the oxidoreductase pathway as depicted in **Supplementary Figure S1**.

### *S. gallolyticus*-Induced Oxidoreductase Expression and Activity

**Figure 2:**
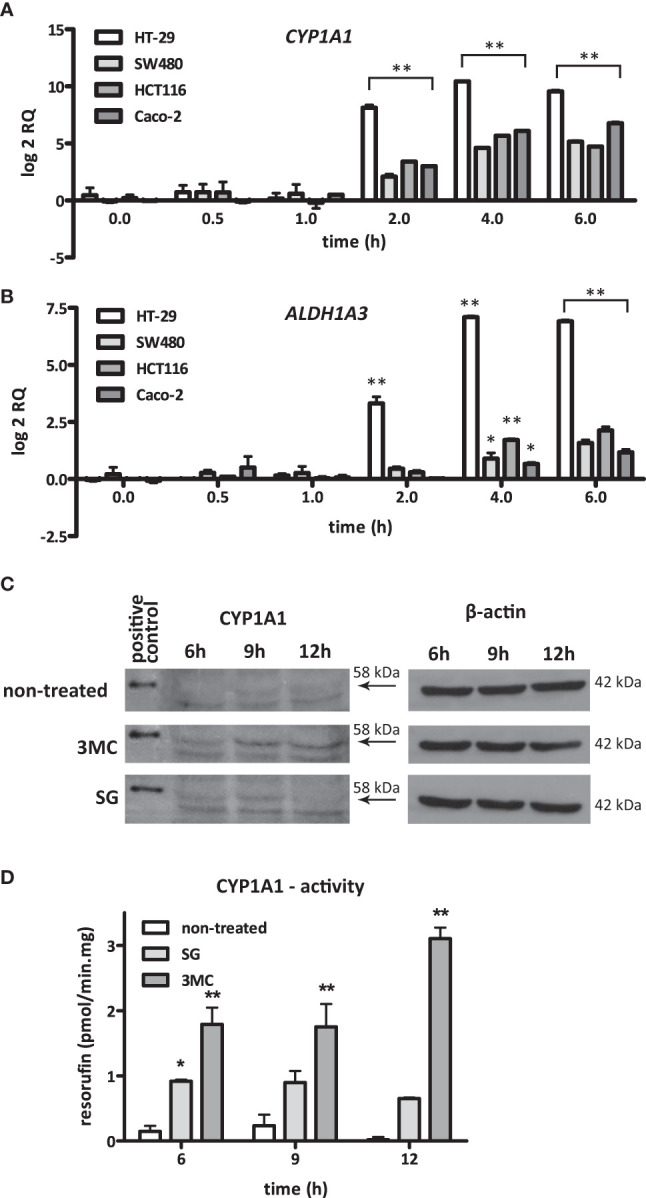
S. gallolyticus-induced CYP1A1 expression. The expression of **(A)** CYP1A1 and **(B)** ALDH1A3 was evaluated by qPCR in the CRC cell lines HT-29, SW480, HCT116, and Caco-2 upon 2, 4, and 6 h of incubation with S. gallolyticus UCN34. Note that the expression of CYP1A1 is highly similar in all investigated cell lines. Two-way ANOVA was performed to determine significant changes (*p < 0.05; **p < 0.01). **(C)** Protein expression of CYP1A1 in Caco-2 cells was investigated by Western blotting after exposure to *S. gallolyticus* or 3MC for 6, 9, and 12 h. The arrows indicate the position of CYP1A1 protein (58 kDa). The replicate blots shown in **Supplementary Figure S3** illustrate that only the indicated band of 58 kDa is detected by several different CYP1A1 antibodies and confirm that only this reactive band specifically appears upon stimulation with 3MC. The right panel shows the corresponding β-actin protein expression levels (42 kDa). **(D)** CYP1A1 enzyme activity was measured by the 7-EROD assay. The bars indicate the amounts of picomoles resorufin produced per minute per milligram protein. Two-way ANOVA was performed to determine significant changes (*p < 0.05; **p < 0.01). RQ, relative quantity; non-treated cells; 3MC, 3-methylcholanthrene; SG, *S. gallolyticus*.

The temporal induction of *CYP1A1* and *ALDH1A3* expression was examined in the cell lines HT29, Caco-2, SW480, and HCT116 after exposure to *S. gallolyticus. CYP1A1* was consistently upregulated (p < 0.001) in all cell lines upon 2, 4, and 6 h of coculturing with *S. gallolyticus* (**Figure 2A**). The induction of *ALDH1A3* was less evident among the different cell lines, although *ALDH1A3* was significantly increased to a log2 RQ between 1.1 for Caco-2 and 6.9 for HT-29 after 4 and 6 h (p < 0.001) (**Figure 2B**). Next, CYP1A1 induction was evaluated at the protein and functional level. To this purpose, Western blot analysis was performed on protein extracts from Caco-2 cells harvested at different time points after *S. gallolyticus* coincubation. Our initial experiments, and the results of others (Meunier et al., 1995), indicated that Caco-2 cells were best suited to detect CYP1A1 at the protein level, and CYP1A1 is only detectably present after stimulation with inducing agents, such as 3-methylcholanthrene (3MC), with maximum protein expression levels after 18–20 h (Rosenberg and Leff, 1993). Because such long incubation times are not feasible with live *S. gallolyticus*, Caco-2 cells were stimulated with *S. gallolyticus* at a high MOI of 50 for 6 h to obtain a short but strong induction, after which bacteria were removed for prolonged incubations of 9 and 12 h (see *Materials and Methods*). 3MC was used in the same time course as a control for CYP1A1 induction in Caco-2 cells (Aboutabl et al., 2009), and human liver microsomes expressing CYP1A1 at high level were loaded as positive control on the Western blot. *S. gallolyticus* induced cellular CYP1A1 levels (~58 kDa) after 6 and 9 h compared to untreated Caco-2 cells (**Figure 2C** and **Supplementary Figure S3**), which was reduced to background levels after 12 h. 3MC continued to increase CYP1A1 levels up to 12 h of stimulation. To validate these findings, CYP1A1 enzyme activity was determined in Caco-2 by the 7-ethoxyresorufin-O-deethylase (7-EROD) assay that measures conversion of ethoxyresorufin into resorufin by functional CYP1A1 enzyme. Resorufin production was found to be significantly increased by *S. gallolyticus* after 6 h incubation (p < 0.05), while 3MC increased CYP1A1 activity at all three time points (p < 0.001) (**Figure 2D**). Thus, these activity data corroborate the real-time and Western blot data that show increased expression of CYP1A1 upon incubation with viable *S. gallolyticus* cells.

### Induction of CYP1 Expression by *S. gallolyticus* Is Dependent on AhR

**Figure 3:**
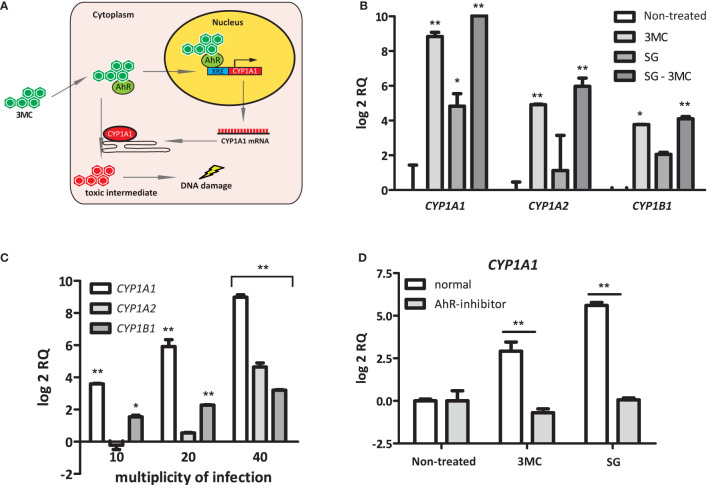
Bacterial Induction of CYP1A1, CYP1A2, and CYP1B1. **(A)** Model for AhR-dependent gene regulation. Polycyclic aromatic hydrocarbons (PAHs) diffuse into the cell and bind to the intracellular Aryl hydrocarbon receptor (AhR). Next, this complex translocates into the nucleus and binds to an XRE-response element, as present in the CYP1A1 promoter region. After transcription/translation, CYP1A1 acts in the endoplasmatic reticulum as a phase I enzyme that converts PAHs to more toxic intermediates that can form DNA adducts. **(B)** CYP1A1, CYP1A2, and CYP1B1 induction was investigated in the presence or absence of the AhR ligand 3MC. Note that *S. gallolyticus* UCN34 (SG) and 3MC have an additive effect on CYP1A1, CYP1A2, and CYP1B1 induction. **(C)** The expression of CYP1A1, CYP1A2, and CYP1B1 upon incubation with *S. gallolyticus* UCN34 at increasing multiplicity of infection was compared to non-treated control cells. **(D)** The induction of CYP1A1 by 3MC or *S. gallolyticus* UCN34 in HT-29 cells, in the presence or absence of an AhR-inhibitor, was investigated by qPCR. Statistical analysis by two-way ANOVA was performed to determine significant changes (*p < 0.05; **p < 0.01).

Polycyclic aromatic hydrocarbon (PAH) substrates, such as 3MC, of CYP1 enzymes interact with the intracellular AhR to induce *CYP1* expression. After the subsequent binding of this AhR-PAH complex to several cofactors, it translocates to the nucleus to activate a xenobiotic response element (XRE) that is present in the promoter regions of the *CYP1A1*, *CYP1A2*, and *CYP1B1* genes (McMillan and Bradfield, 2007) (**Figure 3A**). To examine whether *S. gallolyticus* also targets AhR, the induction of *CYP1A1*, *CYP1A2*, and *CYP1B1* was examined in HT29 cells in combination with 3MC. These experiments showed that *S. gallolyticus* significantly increased *CYP1A1*, *CYP1A2*, and *CYP1B1* and had an additive effect on 0.001 µM 3MC-mediated *CYP1A1* induction (**Figure 3B**; p < 0.05). *S. gallolyticus* also had a slight additive effect on 3MC stimulation for both *CYP1A2* and *CYP1B1* (**Figure 3B**), although this was not significant. Furthermore, *S. gallolyticus* induced *CYP1* expression in a concentration-dependent manner, similar to that of *CYP1A1* (**Figure 3C**; p < 0.001). An AhR antagonist that prevents the binding of PAHs to this receptor (Kim et al., 2006) was added to cocultures to evaluate whether *S. gallolyticus* also depends on AhR. Preincubation of HT-29 cells with this AhR inhibitor clearly blocked the upregulation of *CYP1A1* (**Figure 3D**), *CYP1A2*, and *CYP1B1* (**Supplementary Figure S4**) by both 3MC and *S. gallolyticus*. Taken together, these results show that *CYP1* induction by *S. gallolyticus* is mediated by an AhR-dependent mechanism.

### Increased CYP1 Expression Is Not Specific for *S. gallolyticus*

To investigate whether the induction of *CYP1A1* is a specific feature of *S. gallolyticus*, the expression of this gene was investigated upon coincubation of HT-29 cells with other *S. bovis* and other intestinal bacterial strains. Besides *S. gallolyticus* UCN34, the other *S. bovis* group strains *S. macedonicus* CIP105685T, *S. infantarius* NCTC8133, and *S. gallolyticus* NTB1 and 1293 increased CYP1A1 gene expression (**Supplementary Figure S5A**). From the other intestinal bacteria, *E. faecalis* and *E. cloacae* were strong inducers of *CYP1A1* gene expression in a concentration-dependent manner (p < 0.01) (**Supplementary Figure S5B**). Intermediate activity was observed for *S. lugdunensis*, *E. coli* Nissle 1917, and *S. typhimurium*. Contrarily, coincubation of HT-29 cells with *E. coli* NTB5 did not result in an induction of *CYP1A1* and might even reduce the expression of this gene (**Supplementary Figure S5C**). Moreover, the additive effect of *S. gallolyticus* in combination with 0.001 µM 3MC (**Figure 3B**) was not observed for *E. coli* NTB5 (**Supplementary Figures S5C, D**). Thus, these data show that CYP1 induction is not specific for *S. gallolyticus* and might confer a more general mechanism among a subset of intestinal bacteria.

### A *S. gallolyticus*-Associated Factor Present in the Secretome Induces CYP1A1 Expression

**Figure 4:**
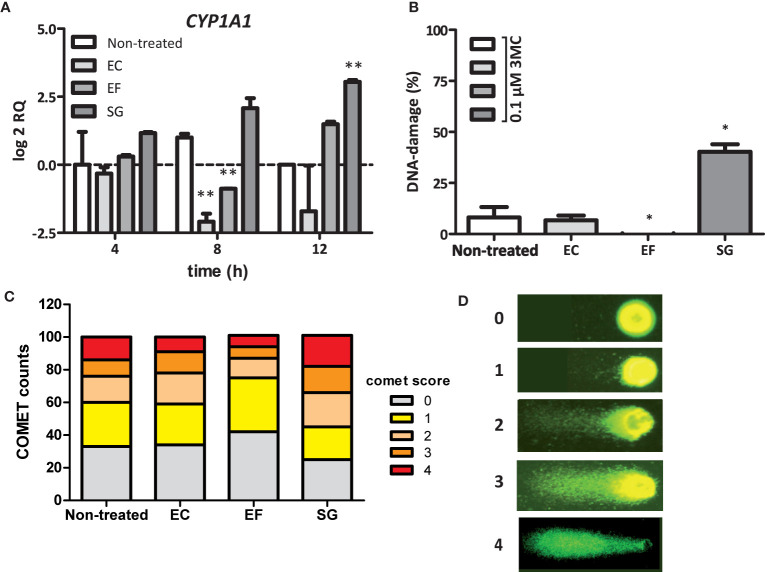
SGS induced CYP1A1 expression and increase in the DNA-damaging effect of 3MC. **(A)** Expression of CYP1A1 in Caco-2 cells was examined by qPCR after exposure of these cells to the secretomes of *E. coli* NTB 5 (EC), *S. gallolyticus* UCN34 (SG), and *E. faecalis* 19433 (EF). Note that only exposure to secretomes from *S. gallolyticus* UCN34 resulted in increased CYP1A1 levels after 8 and 12 h (**p < 0.01). **(B)** DNA damage under the conditions described in Panel **(A)** were measured by the COMET assay (*p< 0.05). To induce low levels of DNA damage, incubation was prolonged for 18 h after addition of 0.1 µM 3MC to the culture medium. Reference ranges were determined by the incubation of Caco-2 cells without 3MC (0% damage) and with 100 µM H_2_0_2_ (100% damage). Only exposure to SGS from *S. gallolyticus* UCN34 in combination with 3MC yielded increased levels of DNA damage compared to 3MC alone (non-treated). (*p < 0.05) **(C)** The increase in DNA damage by SGS in combination with 3MC is mainly due to an increased number of cells with DNA damage and with high levels of DNA damage (COMET scores 3 and 4). Two-way-ANOVA (p < 0.01). **(D)** Representative fluorescence microscope images with assigned COMET scores used for the quantification of DNA damage as shown in **(B, C)**.

A remarkable finding was the fact that the intracellular AhR is stimulated by *S. gallolyticus* cells, which lack the ability to invade CRC cells (Boleij et al., 2011). This suggests that these bacteria release a factor themselves, convert and activate a medium component, or provoke the expression of a diffusible host factor that re-enters CRC cells to induce *CYP1A1* expression. To confirm this hypothesis, secretome from *S. gallolyticus* (SGS) was filtered and added to fresh Caco-2 cells for 4, 8, and 12 h and to HT29-cells for 12 h. As shown in **Figure 4A** and **Supplementary Figure S5E**, SGS induced *CYP1A1* expression in Caco-2 cells and HT29-cells (log2 RQ 3.0; p < 0.01). Interestingly, secretomes from *E. faecalis* only marginally induced *CYP1A1* expression after 12 h (log2 RQ 1.5; NS), whereas viable *E. faecalis* cells strongly induced expression of this gene (**Supplementary Figure S5A**). In line with the previous results, secretomes from *E. coli* NTB5 had no effect on *CYP1A1* expression. Secretomes from other *S. bovis*-group bacteria, such as S. *gallolyticus* subsp. *pasteurianus*, subsp. *macedonicus*, and subsp. *gallolyticus, S. equinus*, or *S. lutetiensus*, were, however, unable to induce *CYP1A1* gene expression (**Supplementary Figure S5E**). These results confirm the idea that a *S. gallolyticus*-associated factor present in the secretome of strain UCN34 induces *CYP1* gene expression through the AhR; however, why secretomes of other strong inducers of *CYP1* are unable to stimulate AhR needs to be resolved.

### SGS Increases the DNA-Damaging Effect of 3MC in CRC Cells

Next, we tested whether CYP1 upregulation by *S. gallolyticus* may contribute to the DNA-damaging effect of 3MC. To test this hypothesis, Caco-2 cells were first incubated in SGS for 6 h to prime CYP1 expression, after which the level of 3MC-induced DNA damage was compared to that in untreated cells by the COMET assay, which quantifies the level of DNA damage on the cellular level. As shown in **Figures 4B–D**, the DNA damaging effect of 0.1 µM 3MC was significantly increased by preincubation of Caco-2 cells in SGS (p < 0.05). In contrast, priming of these cells with secretomes from *E. faecalis* or *E. coli* did not increase 3MC genotoxicity. In fact, secretome from *E. faecalis* had even a surprising inhibitory effect on 3MC-induced DNA damage (p < 0.05). It is presently unknown how this relates to the induction of *CYP1* expression by viable *E. faecalis* cells. Together, these results show that secreted or otherwise released *S. gallolyticus*-associated factors can prime CRC cells towards an increased susceptibility to 3MC-induced DNA damage under the applied experimental conditions.

### No Clear Link Between COX-2 Expression, PGE2 Release, Cell Proliferation, and AhR Activation

**Figure 5:**
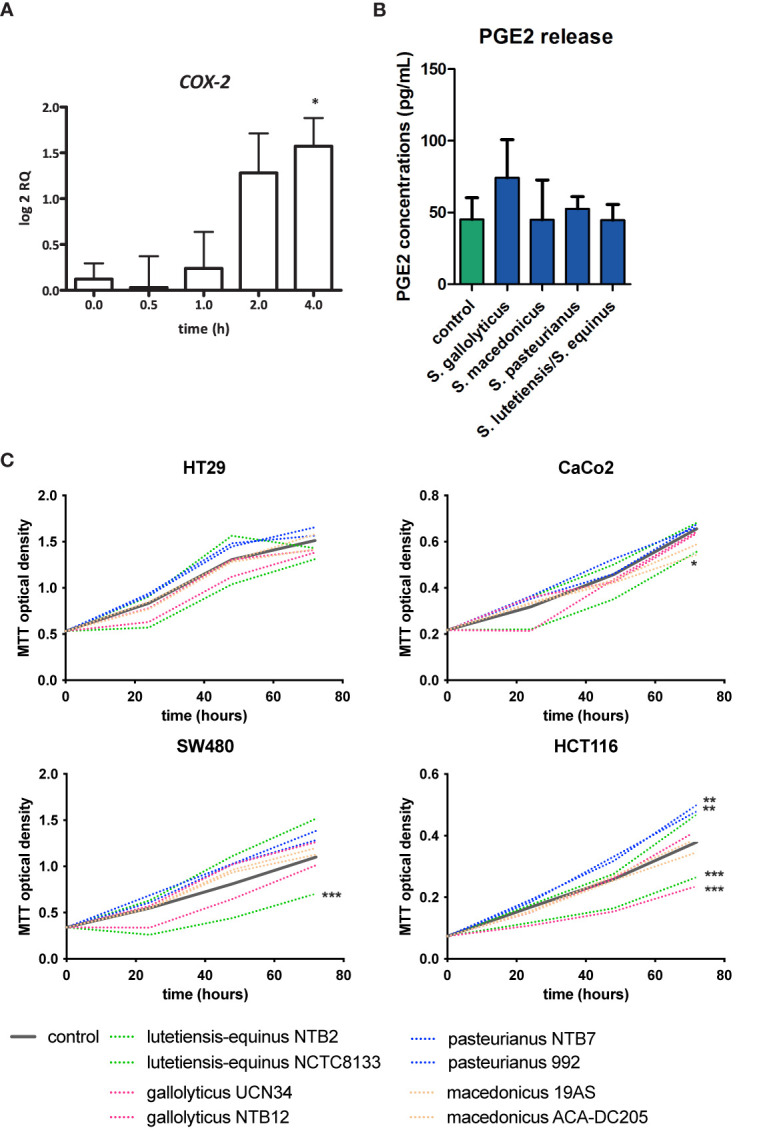
COX-2 induction, PGE2 release, and cell proliferation by SGS. **(A)** Expression of COX-2 in HT29 cells was examined by qPCR after exposure of these cells to *S. gallolyticus* UCN34 at an MOI of 20 for 4 h COX-2 was significantly upregulated as determined by one-way ANOVA (*p < 0.05). **(B)** PGE2 release after exposure to secretomes of *S. gallolyticus* subsp. gallolyticus (UCN34/NTB12), *S. gallolyticus* subsp. pasteurianus (NTB7/992), *S. gallolyticus* subsp. macedonicus (19AS/ACA-DC-205), and S. equinus/lutetiensis (NCTC8133/NTB2). No significant increase in PGE2 release was observed after 24 h. **(C)** Cell growth was measured with MTT assay at 24, 48, and 72 h in HT29, Caco-2, HCT116, and SW480 cells. Each secretome condition was performed in quadruplicate. The area under the curve was calculated for each condition and compared to control cells using independent t-test. HCT116 cells were most sensitive to S. bovis group bacteria. In none of the conditions, *S. gallolyticus* subsp. gallolyticus secretomes induced cell growth. *S. gallolyticus* subsp. gallolyticus NTB12 even inhibited cell growth in HCT116 cells. Only *S. gallolyticus* subsp. pastereurianus strains 992 and NTB7 were able to induce cell growth in HCT116 cells. S. lutetiensis NTB2 consistently inhibited cell growth in CaCo-2, SW480, and HCT116 cells. *p < 0.05, **p < 0.01, ***p < 0.001.

It was previously shown that the related bacterium *S. infantarius* NCTC 8133, now reclassified as *S. equinus*, induced COX-2 expression in colon epithelial cells *in vitro* (Ellmerich et al., 2000). Moreover, it has been described that COX2 expression can, similarly to CYP1A1, also be activated *via* AhR-ligand binding to the XRE promotor (Degner et al., 2007). Therefore increased AhR activity and CYP1A1 might also relate to COX-2 upregulation. *S. gallolyticus* UCN34 showed a low, but significant, induction of *COX-2* expression to a log2 RQ of 1.6 in HT-29 cells after 2 and 4 h (p = 0.017) of coculturing (**Figure 5A**). However, at protein level, PGE2 release was not significantly increased in HT29 cells compared to control cells after 24 h exposure to SGS (74.2 *vs.* 45.0 pg/ml, p > 0.05, **Figure 5B**). Furthermore, it has been described that AhR activation may lead to cell proliferation. Previously, it was shown that *S. gallolyticus* increases cell proliferation of several colon epithelial cell lines, dependent on the bacterial strain (Kumar et al., 2018). We show here that at least SGS of strain UCN34 and NTB12 are unable to increase cell proliferation in HT29, SW480, HCT116, and Caco-2 cells. NTB12 even decreased cell proliferation significantly in HCT116 cells (**Figure 5C**). Similarly, *S. lutetiensis* NTB2 consistently decreased cell proliferation in Caco-2, SW480, and HCT116 cells. Only *S. gallolyticus* subsp. *pasteurianus* NTB7 and 992 were significantly increasing growth of HCT116 cells (p < 0.001). Hence, a clear correlation between secretomes, AhR-activation, cell proliferation, and PGE2 release is not observed from these results.

### *S. gallolyticus* Increases CYP1A1 Expression *In Vivo*

**Figure 6:**
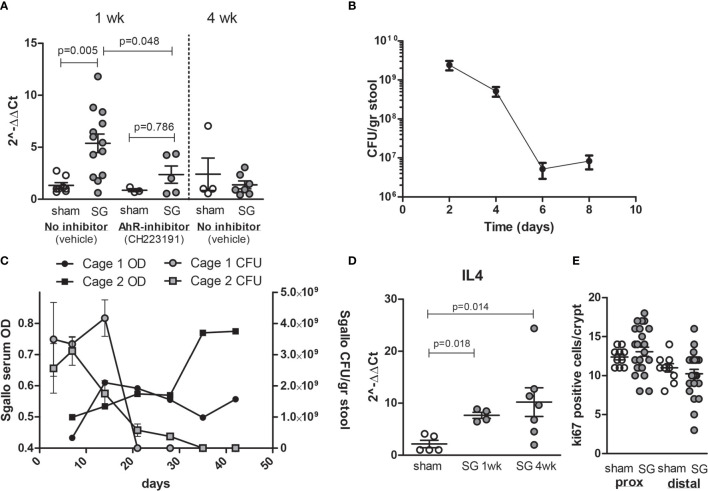
Colonization of C57bl6 mice leads to CYP1A1 induction in cecum and effective clearance of *S. gallolyticus*. **(A)** Compared to sham mice (n = 8), S. galloltyicus UCN34 colonized mice show significantly increased levels of CYP1A1 at 1 week postcolonization (n = 13, Mann–Whitney U-test p = 0.005) that is not observed in mice treated with daily gavage of the AhR-inhibitor CH223191 (n = 5). At 4 weeks postcolonization, CYP1A1 induction is back to normal levels (n = 7). **(B)** Stool colonization of UCN34 monitored at 2 (n = 4), 4 (n = 8), 6 (n = 9), and 8 days (n = 14) postcolonization. Two out of five AhR-treated animals lost colonization by day 8, whereas 3 out of 13 vehicle-treated-mice lost colonization. The colonization levels at days 6–8 were not significantly different for vehicle or AhR-treated animals (Mann–Whitney U-test, p = 0.12, **Supplementary Figure S6**). **(C)** Colonization of S. galloltyicus UCN34 over time up to 42 days (6 weeks) with weekly serum collections (without vehicle or AhR-inhibitor) (n = 8). CFU of S. galloltyicus in gray circles (cage 1; n = 4) and squares (cage 2;n = 4) decreased after 3 weeks significantly, while antibody production starts to increase at 2–3 weeks postcolonization (black circles and squares). Cages were visualized separately because of differences in colonization and antibody responses between the two cages. The treatment, inoculation, and handling of the animals in these two cages was similar. **(D)** IL4 mRNA production is increased at 1 and 4 weeks post-UCN34 colonization compared to sham mice (Mann–Whitney U-test p = 0.018 and 0.014, respectively). **(E)** ki67 stained slides were scored for total number of positive ki67 cells per crypt; for each mice, five crypts were counted and plotted. No difference in ki67-positive cells was seen in proximal and distal colon at 1 week postcolonization compared to sham.

To verify that CYP1A1 activation *via* AhR in *in vitro* cell models is also observed *in vivo*, wild-type C57bl6 mice were colonized with *S. gallolyticus* UCN34 after 5 days of antibiotic treatment with kanamycin/doxycyclin. Mice were either pretreated with vehicle or with daily gavage of 10 mg/kg AhR inhibitor CH223191. After 1 week of colonization, a clear induction of CYP1A1 mRNA was detected in the cecum of mice colonized with *S. gallolyticus*, which was significantly reduced in mice fed AhR inhibitor (**Figure 6A**). Mice colonization was measured in fecal pellets with levels of 1-3*10^9^ CFU/g stool at 3 days postcolonization waning to levels of 1–10 × 10^7^ CFU/g stool at 1 week (**Figure 6B** and **Supplementary Figure S6**). As levels of *S. gallolyticus* started to drop at 1 week, we tested how long colonization would persist and whether effective immune response would eliminate *S. gallolyticus*. We observed colonization up to 21–30 days after which *S. gallolyticus* in stool was below the detection level. Simultaneous with reduced colonization levels, IgGs detecting immobilized *S. galloltyicus* in an ELISA assay were present in serum of mice colonized with *S. galloltyicus* (**Figure 6C**). To confirm that a B-cell response was initiated, IL4 mRNA was measured and detected at 1 week with increasing levels 4 weeks postcolonization (**Figure 6D**). CYP1A1 was no longer increased at 4 weeks postinoculation when colonization of *S. gallolyticus* disappears. In addition, *in vivo*, we did not observe any increase in prostaglandin expression at mRNA and protein level (data not shown) or differences in cell proliferation in proximal or distal colon (**Figure 6E** and **Supplementary Figure S7**). The cecum showed some immune cell infiltration and reactive epithelial changes with *S. gallolyticus* colonization (**Supplementary Figure S8**), but this was not significantly different from sham mice, and no differences were observed between mice treated with vehicle or AhR inhibitor. No inflammation or aberrant growth was observed in the cecum or colon. Together, these data show that also *in vivo* CYP1 is induced by *S. gallolyticus* and that *S. gallolyticus* is effectively recognized and eliminated by the mouse intestinal immune system.

## Discussion

Accumulating evidence supports a relationship between intestinal bacteria and the pathogenesis of CRC (Wang et al., 2008; Wu et al., 2009; Cuevas-Ramos et al., 2010; Rubinstein et al., 2019; Dziubanska-Kusibab et al., 2020; Pleguezuelos-Manzano et al., 2020). For *S. gallolyticus*, the clinical relation with CRC is unambiguous. Detection of *S. gallolyticus* in blood is an indication for colonoscopy (Boleij et al., 2011); there is an increased colonization reported in CRC patients (Jans and Boleij, 2018), and in large seroepidemiological studies, a significant exposure to *S. gallolyticus* antigens in serum was found in CRC patients (Boleij et al., 2012; Butt et al., 2016; Butt et al., 2017; Butt et al., 2019). However, it is still debated whether *S. gallolyticus* merely profits from the tumor microenvironment and/or whether *S. gallolyticus* can also contribute to CRC development. The aim of this study was to find *S. gallolyticus-*induced pathways in CRC cells. By a microarray profiling approach of *in vitro*-cultured CRC cells, we discovered that *S. gallolyticus* is a potent inducer of cellular biotransformation enzymes CYP1 and ALDH1. In particular, our data show that *S. gallolyticus* induces CYP1 enzyme production through AhR by a component or components present in the secretome (SGS) that remains to be identified. Clearly, SGS increases the DNA damaging effect of 3MC in CRC cells, showing that *S. gallolyticus* is somehow able to functionally alter the biotransformation capacity of Caco-2 cells *in vitro* and also induces the same pathway through AhR *in vivo* in wild-type C57bl6 mice.

The expression of CYP1 is induced by their toxic substrates, mainly polycyclic aromatic hydrocarbons (PAHs) present in charbroiled food, environmental pollutants accumulating in the food chain, such as digoxin, and cigarette smoke. These toxic substrates bind to AhR that translocates to the nucleus and activates transcription of genes with an AhR response element such as CYP1 and ALDH (Dietrich and Kaina, 2010). Depending on the substrate, induction of biotransformation enzymes contributes to either the detoxification or bioactivation of these toxins and thereby modulate the carcinogenic potential of these compounds (Shimada et al., 1992; Kim et al., 2009). CYP1 enzymes oxidize or hydrolyze (toxic) compounds (phase I metabolism) often resulting into more reactive intermediates (Eastman and Bresnick, 1979), a process that is also called bioactivation. This is often followed by conjugation reactions catalyzed by phase II enzymes, such as glutathione S-transferases (GSTs) or UDP-glucuronosyltransferases (UGTs) to deactivate these compounds and make them more water soluble for clearance from the body. Biotransformation of PAHs by CYP1 often results in chemical intermediates that are more genotoxic than their precursors. These intermediate carcinogens can covalently bind to chromosomal DNA that interferes with correct DNA replication and in turn may result in increased mutation rates (Eastman and Bresnick, 1979; Spink et al., 2002). Induction of CYP1 enzymes is mediated through the AhR that can interact with a wide range of structurally diverse ligands (Denison and Nagy, 2003; Abel and Haarmann-Stemmann, 2010). Here, we show that a multitude of intestinal bacteria can induce CYP1, as exemplified by *S. gallolyticus*. CYP1 is inhibited *in vitro* and *in vivo* when blocking the AhR with CH223191. Induction of phase I enzymes may result in the accumulation of toxic intermediates, which is corroborated by the observation that priming CRC cells with secretomes of *S. gallolyticus* results in the increased formation of DNA damage upon exposure of these CRC cells to 3MC. 3MC is converted into more carcinogenic intermediates such as dihydrodiol- or 2-hydroxy-3MC that has higher DNA-binding efficiencies than the parent compound 3MC. These bioactivation events thereby result in increased DNA adduct formation and consequently higher mutation rates (Eastman and Bresnick, 1979). However, from these results, we cannot firmly conclude that the potentiation of 3MC by secretomes of *S. gallolyticus* depends on AhR activation. Other *S. gallolyticus*-associated factors, which act in an AhR-independent manner, may also add to the observed increase in DNA damage. Nonetheless, our current *in vitro* experiments show the potency of a multitude of bacteria to modulate CYP1 within CRC cells.

*In vivo*, the activation of the AhR contributes to the regulation of cell growth *via* a negative feedback mechanism mediated by the AhR repressor (AhRR) (Hahn et al., 2009). It was previously shown that AhRR expression levels are modestly decreased in precancerous colonic polyps and more profoundly decreased in primary invasive colon carcinomas (Zudaire et al., 2008). This underscores the importance of AhR stimulation during malignant transformation. Besides this, it has been shown that CYP1 enzymes are poorly expressed in healthy colonic epithelium but overexpressed in colonic adenomas and carcinomas (McKay et al., 1993; Mercurio et al., 1995; Ding and Kaminsky, 2003). Our current data show that bacteria have the potency to interfere with AhR-mediated induction of CYP1A1 *in vitro* and *in vivo* and thereby could modulate important cellular processes, such as carcinogenesis of food-derived PAHs.

Our *in vivo* data show that colonization with *S. gallolyticus* is only present 3 weeks postinoculation and exerts an effective antibody response in the serum of the mice. In line with this observation, *S. gallolyticus* disappears from the colon, and CYP1 induction in the ceca of the mice drops back to normal levels. This suggest that active colonization is required for the *in vivo* observed induction of biotransformation ability by CYP1. Whether this induction *in vivo* also contributes to increases in DNA damage by PAHs has not been investigated yet. Alternatively, AhR activation in intestinal epithelial cells has also been linked to intestinal barrier function and an effective immune response (Yu et al., 2018). Hence, the activation of AhR by (opportunistic) pathogens *in vivo* might have a dual role and also play a role in effective protection against pathogens. Alternatively, AhR deficiency *in vivo* has been linked to chemical-induced tumorigenesis in infection models with DSS and *Citrobacter rodentium* (Diaz-Diaz et al., 2016; Metidji et al., 2018). In the latter model, AhR was shown to negatively regulate the Wnt-β-catenin pathway and prevent the development of tumors by AOM. While *S. gallolyticus* secretomes may potentiate 3MC *in vitro* in epithelial cells without affecting cell proliferation, the outcome *in vivo* might depend on the level of AhR stimulation and cell proliferation by *S. gallolyticus* and should be further investigated.

Unfortunately, until now we were unable to identify the molecular structure of the *S. gallolyticus*-associated factor(s) that mediate increased CYP1 expression in CRC cells. Candidates are shed structural components of the bacterial surface or excreted metabolites. Alternatively, this factor may be formed by *S. gallolyticus* from a precursor that is present in the growth medium. As killed *S. gallolyticus* did not induce *CYP1* expression, we consider the latter two options as most likely. A possible mechanism could be the bacterial conversion of tryptophan into indoles (Nebert and Karp, 2008), or the production of short-chain fatty acids (Jin et al., 2017), which are known ligands of AhR. Alternatively, *S. gallolyticus* may induce the production or release of a factor from CRC cells, which can (re)enter CRC cells. Clearly, future biochemical studies are required to identify the *S. gallolyticus*-associated or more broadly intestinal bacteria-associated factors, which can induce CYP expression in an AhR-dependent manner.

It should be realized that a potential caveat of our study is that only transformed CRC cell lines were used, in which signaling pathways are already different from normal epithelial cells. Unfortunately, our attempts to confirm our data in primary healthy colonic cells were unsuccessful due to difficulties in maintaining their viability during the experimental conditions of this study. However, *S. gallolyticus* could effectively induce CYP1 expression in healthy C57bl6mice, which indicates that AhR activation is not a sole feature of CRC cells.

The increased colonization of *S. gallolyticus* observed with CRC was recently confirmed in APC^min^ and APC/notch mice, where a clear increase in colonization was reported, but no increase in tumor burden with *S. galloltyicus* subp. *gallolyticus* UCN34 was observed (Aymeric et al., 2018). However, Kumar et al. showed that tumor burden and cell proliferation depended on the *S. gallolyticus* strain used *in vitro* and *in vivo*, which was observed for strain TX20005 but not TX20008 (Kumar et al., 2018). Our data support these findings; *S. gallolyticus* UCN34 was effectively cleared from wild-type C57bl6 mice and did not increase cell proliferation *in vitro* and *in vivo*. Furthermore, our unpublished pilot in APC^min^ mice at 6 weeks shows no increase in tumor burden in these susceptible mice upon *S. gallolyticus* UCN34 colonization. These strain level effects on cell proliferation for a multitude of CRC-associated bacteria were recently also shown by us for *Fusobacterium nucleatum* and *Clostridium* species (Taddese et al., 2020). These combined data suggest effective long-term colonization with *S. gallolyticus* only when tumors are present and, more importantly, that strain level differences are important for the observed effects.

In conclusion, our data provide intriguing evidence for the bacterial potential, especially *S. gallolyticus* subsp. *gallolyticus*, to interrelate with AhR-mediated pathways for cellular biotransformation (CYP1) that could potentially contribute to DNA damage and/or is involved in effective intestinal clearance of (opportunistic) pathogens protecting the epithelial barrier. To confirm the AhR-mediated effects *in vitro*, AhR reporter cell lines could aid in understanding which bacteria or which component in the secretome exert the induction of CYP1 genes. Furthermore, *in vivo*, the role of *S. gallolyticus* or related bacteria on epithelial and immune compartment could be further explored by the use of AhR knockout-mice crossed with villin-cre or bone-marrow chimera models with AhR knockout and wild-type mice to deduct the role of epithelial and immune cells in the *S. gallolyticus* AhR interactions. When the component in the secretome is known, also titration studies could shed light on the impact of such compound *in vivo*. Our data underscore the need for a better understanding of host–microbe interactions during CRC, since it builds on accumulating studies that implicate bacteria in the initiation and progression of CRC. Emerging studies moving from association to causation in microbiome research will in the near future further shed light on species and even strain-dependent actions of microbiota on CRC development that, in our opinion, are increasingly necessary due to species- and strain-dependent effects; e.g., not all *Streptococci* are equal.

## Data Availability Statement

The datasets presented in this study can be found in online repositories. The names of the repository/repositories and accession number(s) can be found below: https://www.ncbi.nlm.nih.gov/geo/, GSE29295.

## Ethics Statement

The animal study was reviewed and approved by the Johns Hopkins University Animal Care and Use Committee.

## Author Contributions

AB, RT, RR, and DD performed and conceptualized *in vitro* experiments. AB, XW, and SW performed and conceptualized mice experiments. AB, HT, and DS conceptualized the study and were responsible for study design and preparation/revision of the manuscript. All authors contributed to the article and approved the submitted version.

## Funding

AB was supported by the Dutch Cancer Society (KWF; project KUN 2006-3591), NWO Rubicon grant 825.11.031, and Veni grant 016.166.089; RR was supported by the Dutch Digestive Diseases Foundation (MLDS; project WO 10-53); and RT was supported by the RIMLS grant 014-058.

## Conflict of Interest

The authors declare that the research was conducted in the absence of any commercial or financial relationships that could be construed as a potential conflict of interest.

## Publisher’s Note

All claims expressed in this article are solely those of the authors and do not necessarily represent those of their affiliated organizations, or those of the publisher, the editors and the reviewers. Any product that may be evaluated in this article, or claim that may be made by its manufacturer, is not guaranteed or endorsed by the publisher.
