# Supplementary material for: Streptococcus gallolyticus Increases Expression and Activity of Aryl Hydrocarbon Receptor-Dependent CYP1 Biotransformation Capacity in Colorectal Epithelial Cells
Source: Front Cell Infect Microbiol. 2021 Oct 27;11:740704. doi: 10.3389/fcimb.2021.740704 (PMC8579041; doi:10.3389/fcimb.2021.740704)
Supplement: Supplementary file 1 [file DataSheet_1.pdf]

Supplementary data:

Streptococcus gallolyticus increases expression and activity of Aryl Hydrocarbon Receptor-dependent CYP1 biotransformation capacity in Colorectal Epithelial Cells

Rahwa Taddese[1], Rian Roelofs[2], Derk Draper[1], Xinqun Wu[4], Shaoguang Wu[4], Dorine W. Swinkels[2], Harold Tjalsma[2] and Annemarie Boleij*[1]

Departments of [1] Pathology, [2]Laboratory Medicine, Nijmegen Institute for Molecular Life Sciences (RIMLS), of the Radboud University Medical Centre (Radboudumc),[4]Johns Hopkins University, Department of Medicine, Division of Infectious Diseases, Baltimore MD, United States of America.

***Corresponding author:** Mailing address: Department of Pathology hp824, Radboud University Medical Centre, P.O. Box 9101, 6500 HB Nijmegen, the Netherlands. Phone: +31-615257548. E-mail: Annemarie.Boleij@radboudumc.nl

SUPPLEMENTAL FIGURES

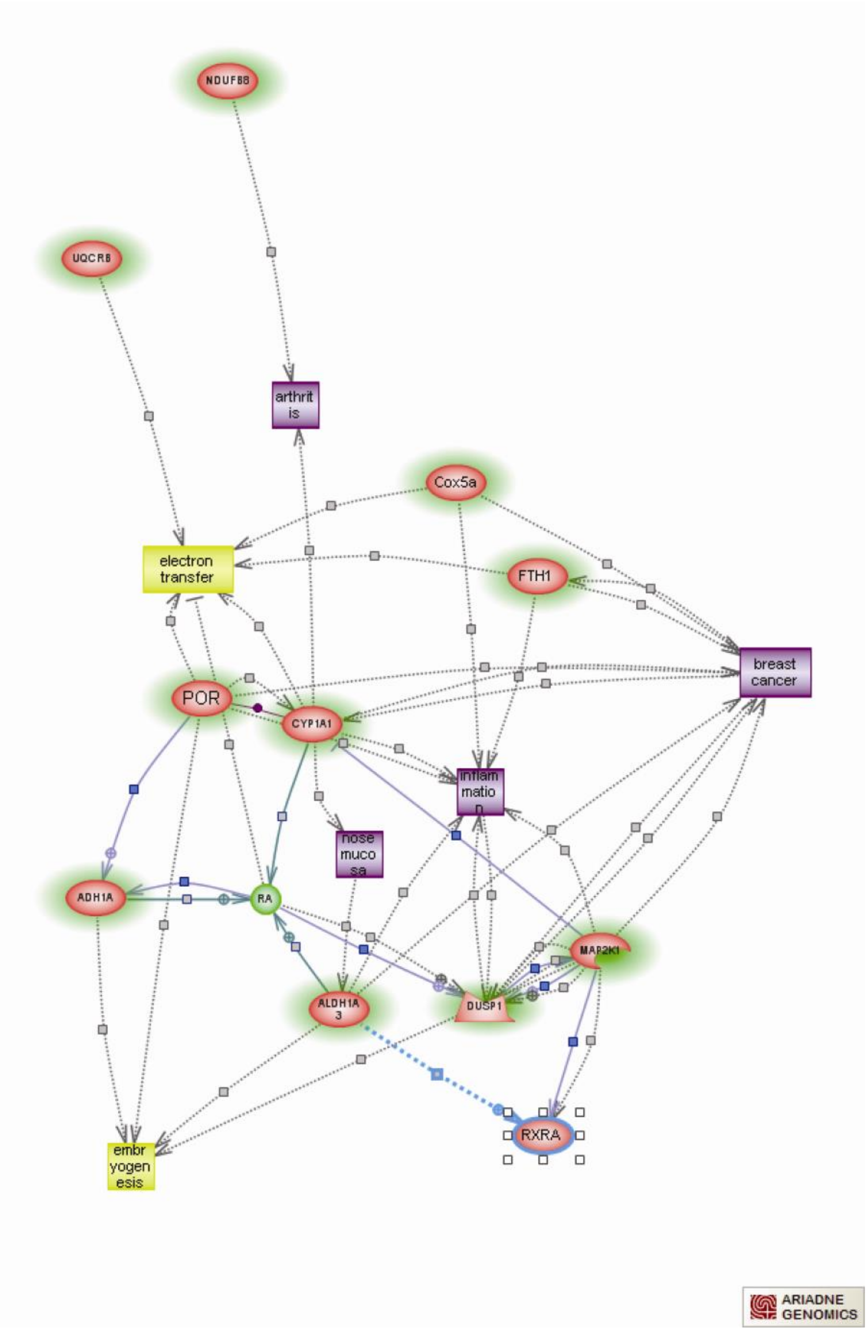

Supplementary figure S1. Oxidoreductase pathway. All significantly up- and down-regulated genes in the microarray, according to the criteria described in the main article, were mapped to known pathways with Ariadne genomics (http://www.ariadnegenomics.com/products/pathway-studio/). All pathways with more than 1 hit were retrieved. This revealed that 9 of the 44 identified genes that were significantly altered upon incubation with *S. gallolyticus* UCN34 related to the oxidoreductase pathway, including CYP1A1, ADH1A and ALDH1A3. Genes are presented in ovals and processes/states in squares. Lines represent the links between genes and processes/states.

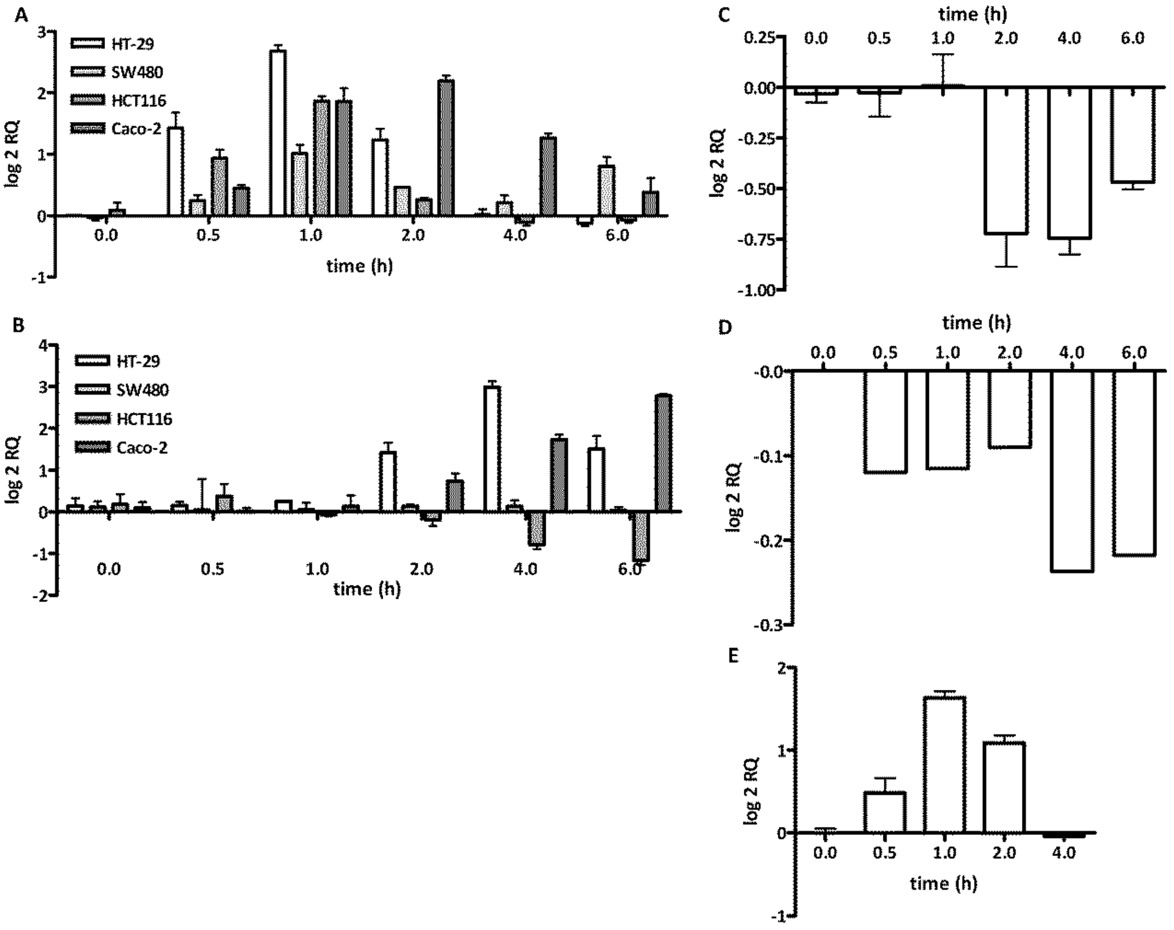

Supplementary figure S2. Gene expression validation of additional genes. DUSP-1 (A) and NPPC (B) gene expression in HT-29, SW480, HCT116 and Caco-2 adenocarcinoma cells was evaluated with Q-PCR upon co-culturing with *S. gallolyticus* UCN34 for the indicated time periods. ZNF561 (C), POR (D) and c10orf10 (E) gene expression was only validated in HT-29 cells. All gene expression levels corresponded with micro-array analysis after 4 hours

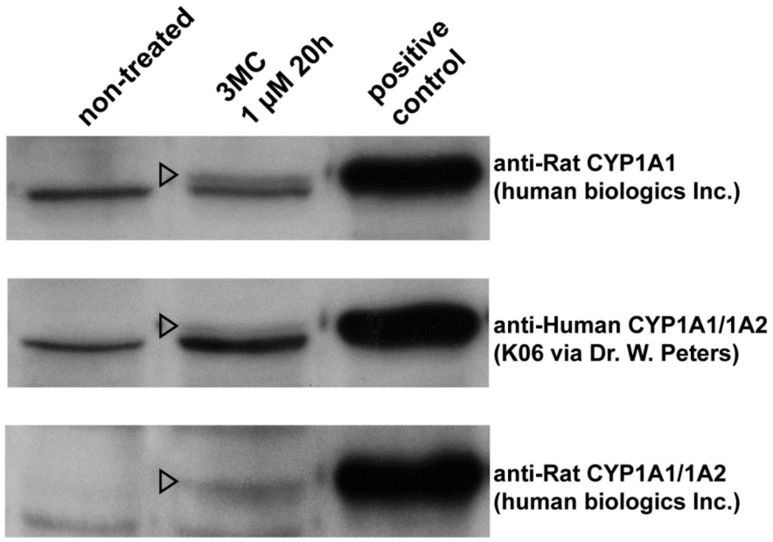

Supplementary figure S3. Western blots probed with CYP1 antibodies. Caco-2 cells were non-treated (lane 1) or incubated with 1µM 3MC (lane 2) for 20 hours. Three different CYP1 antibodies (indicated at the right side of the panels) all detected the same protein band of 58 kDa in replicate blots (indicated by arrows). A human microsomal liver fraction was used as positive control for CYP1 (lane 3). Unfortunately, none of the tested antibodies was highly specific for CYP1A1. Eventually, monoclonal mouse anti-CYP1A1 antibodies (Santa-Cruz) were selected for use in the *S. gallolyticus* UCN34 co-incubation experiments (Figure 2C of the main article) as these had a similar sensitivity as the antibodies shown above, but yielded a clear separation between the CYP1A1 signal and co-reacting bands

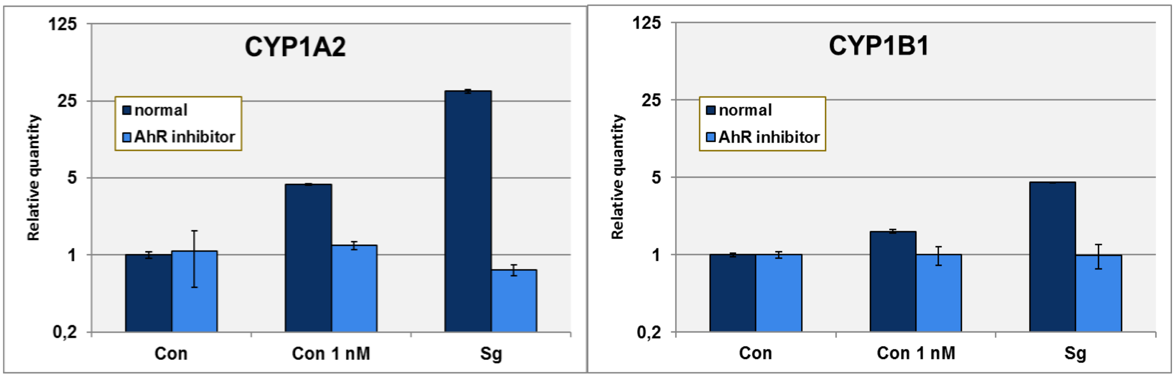

Supplementary figure S4 CYP1A2 and CYP1B1 inhibition by the AhR-inhibitor. Induction of CYP1A2 and CYP1B1 that are also regulated by the AhR-receptor is also inhibited by the AhR-inhibitor both in 3MC treated cells (Con 1nM) and in *S. gallolyticus* UCN34 treated cells.

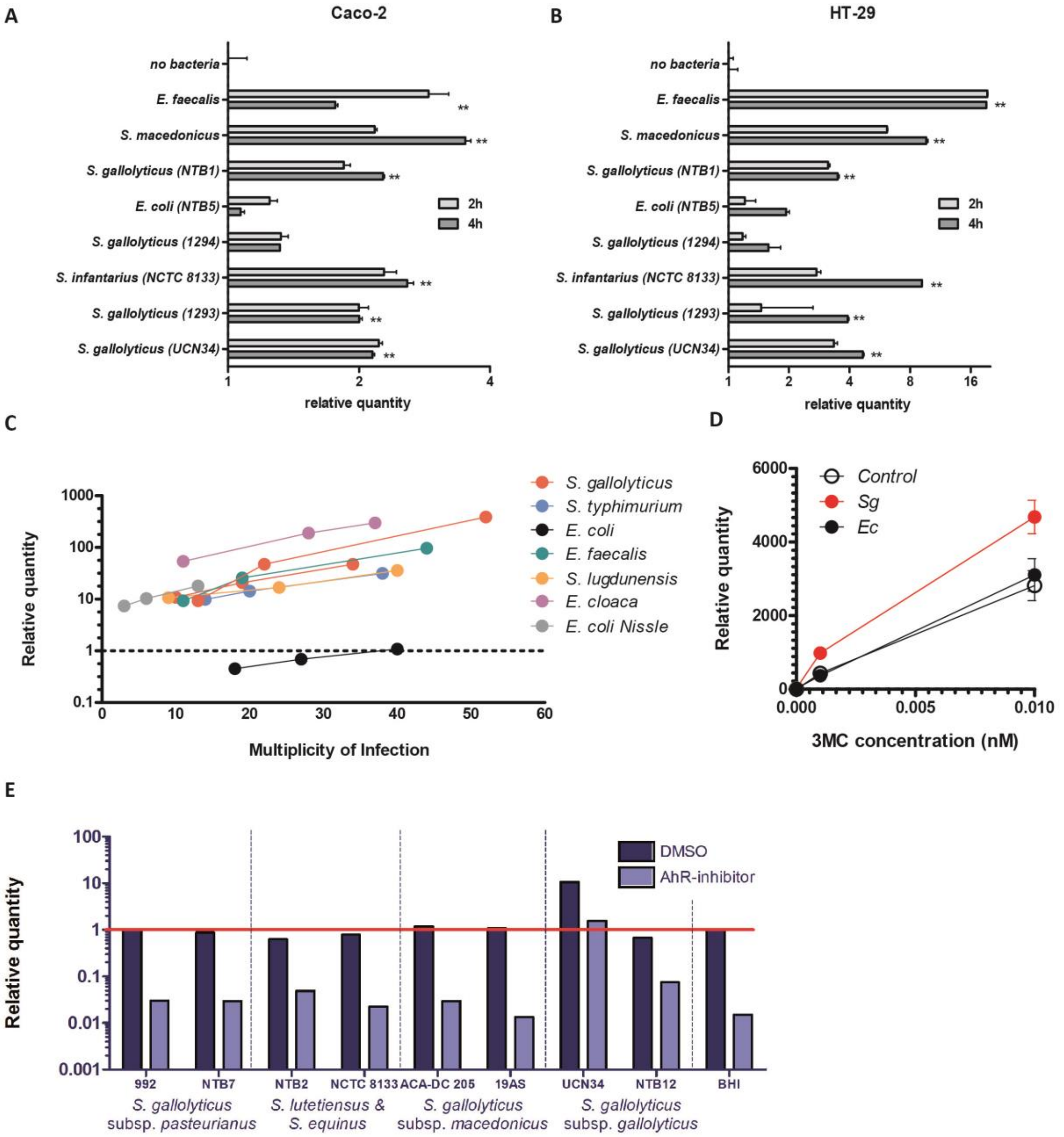

Supplementary figure S5. CYP1A1 induction by other *S. bovis* bacteria. HT-29 (A) and Caco-2 (B) cells were incubated with the indicated bacteria at an MOI of 20 for 2 and 4 hours. Delta-delta Ct values are presented at the X-axis. (Two-way ANOVA * $p<0.05$, ** $p<0.01$). All bacteria, except *E. coli* NTB5 and *S. gallolyticus* subsp. *gallolyticus* 1294 were able to induce CYP1A1 at the mRNA level in HT-29 and Caco-2 cells. (C) Several intestinal bacteria were incubated with HT29-cells and increased CYP1A1 expression in a concentration dependent manner. Only *E. coli* NTB5 did not induce CYP1A1 expression. (D) *S. gallolyticus* UCN34 showed an additive effect on CYP1A1 expression in combination with 3MC compared to *E. coli* NTB5 and control cells. (E) Whereas several S. bovis group strains induced CYP1A1 expression with live bacterial cells (figure A and B), only *S. gallolyticus* UCN34 induced CYP1A1 expression while exposing HT29 cells to bacterial secretomes, which was inhibited by the AhR-inhibitor.

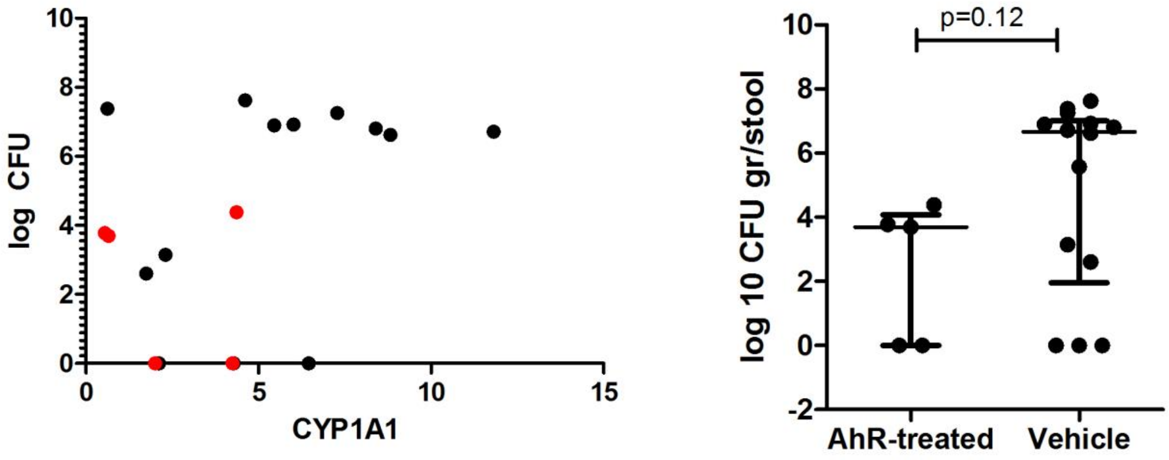

Supplementary figure S6. ***S. gallolyticus*** **colonization per CYP expression and AhR-treatment.** A) CFU/gr stool colonization with S. gallolyticus UCN34 at day 6-8 compared to the levels of CYP1A1 expression. Mice treated with the AhR-inhibitor are depicted with red dots. In total 2 out 5 AhR-treated mice lost colonization at day 6-8, while 3 out of 13 vehicle treated mice lost colonization at days 6-8. B) The median CFU/gr stool was not significantly different between AhR-treated and vehicle treated mice (Mann-Whitney U-test, p=0.12)

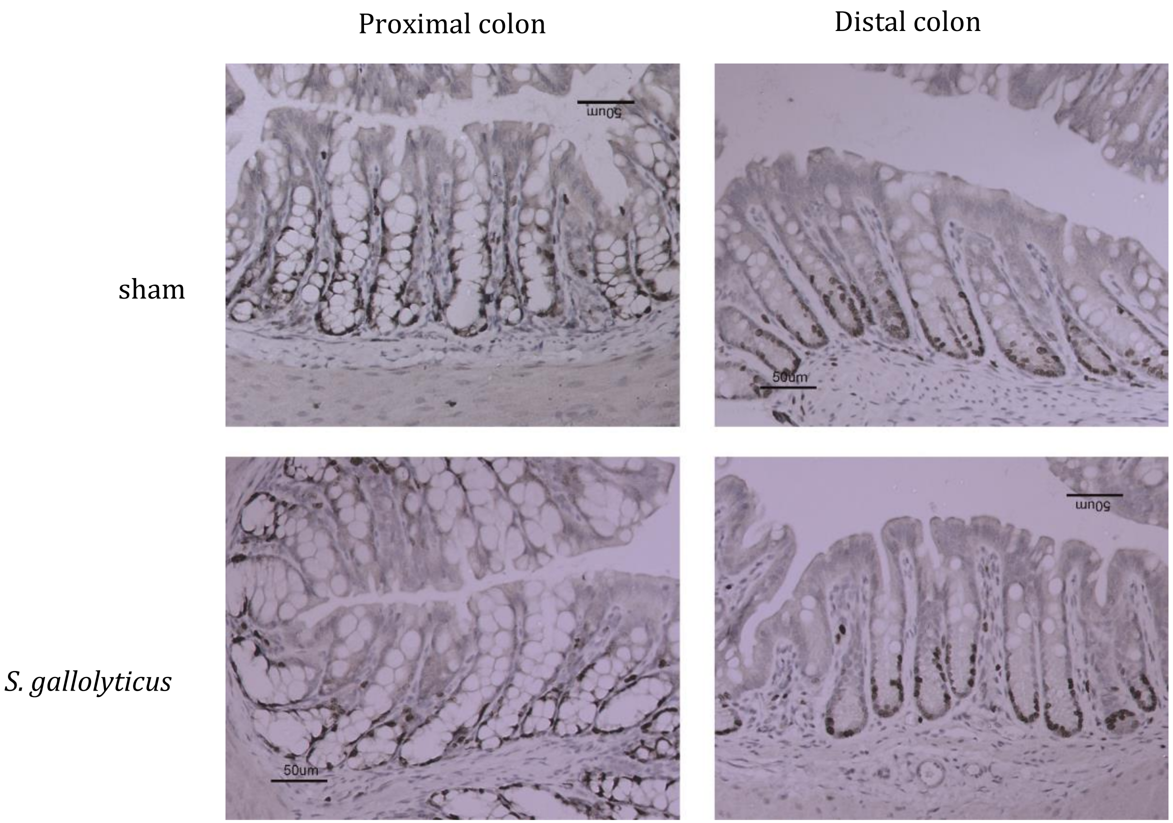

Supplementary figure S7. Ki67 stainings of proximal and distal colon.
Representative pictures of ki67 stainings in proximal and distal colon of sham and *S. gallolyticus* UCN34 treated mice at 1 week (pictures taken at 20x).

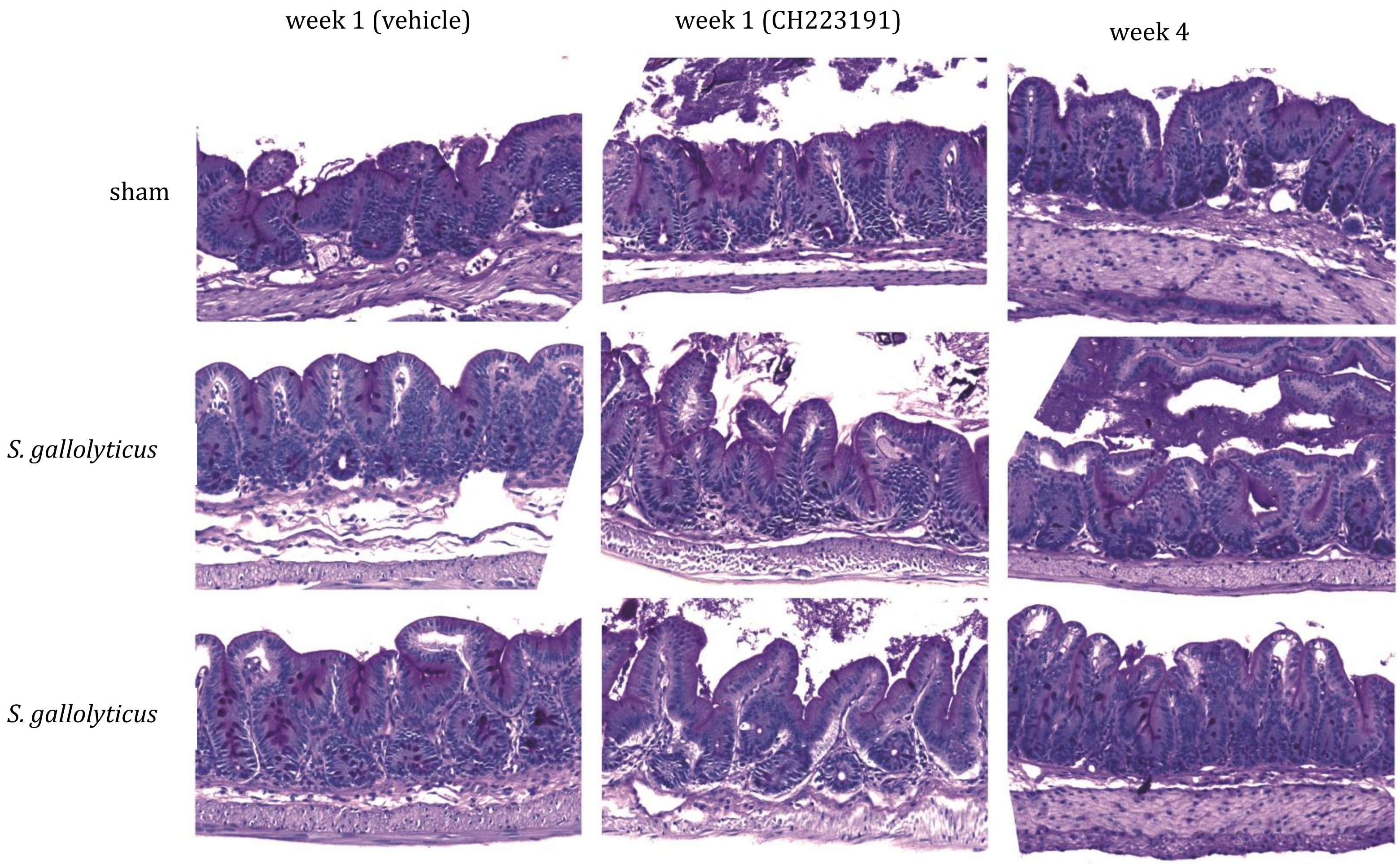

Supplementary figure S8. PAS stainings of cecum.
Representative pictures of two *S. gallolyticus* UCN 34 and 1 sham treated mice at 1 week without or with AhR-inhibitor CH223191, and at 4 weeks post-colonization. Pictures are taken at 20x. Some immune cells are observed in the lamina propria in *S. gallolyticus* UCN34 treated mice, but no clear differences are observed between sham and *S. gallolyticus* UCN34 treated mice. Histology was judged with an expert GI pathologist.

Supplemental Table S1. Primers and gene-expression assays

Gene	Forward primer	Reverse primer	Gene-expression assay
C10orf10			Hs00199735_m1
DUSP1	cgaggccattgacttcataga	ctggcagtggacaaacacc	
ZNF561	agtgcggtttcgcctttat	agggttatttgcggtccac	
POR	aggtgtacatgggggagatg	gaacggattcttggcatcaa	
ZSCAN20	gttgtgaagtgggtgtctcg	acggtgagtccttcaagagg	
NONO	ctggacagatgcagtgaagg	cacagtcacaggacgaggaa	
ULBP3	aggaagaagaggctggaacc	ctatggctttgggttgagcta	
RAET1L	catcccagctttgcttctgt	gcaaagagagtgagggtcgt	
C3orf19	cgacgatgaggaaaaccttc	ccaaagagtccacgtaatcca	
TRUB1	agctgctgaatcggttgaag	ttcctcttggtccattctgg	
LYPLA2	caagtacatctgtccccatgc	gactcagccccatcaggtc	
COX5A	caaagtgtaaaccgcatgga	tccaggtaactgttcacactcaa	
ADH1A	caccagtctcctggtctgc	cagctgctttgcatttgatt	
FTH1	gaagctgcagaaccaacga	cacactccattgcattcagc	
MAP2K1	cattgctgtaataaaaggcctga	tgttggagggcttgacatc	
NPPC			Hs00360930_g1
ALDH1A3	tctcgacaaagccctgaagt	ggcgttgtagcagttgatcc	
CYP1A1			Hs00153120_m1
CYP1A2			Hs01070374_m1
CYP1B1			Hs00164383_m1
PTGS2 (COX-2)			Hs00153133_m1
